# Reducing the Toxicity of Long-Term Glucocorticoid Treatment in Large Vessel Vasculitis

**DOI:** 10.1007/s11926-020-00961-0

**Published:** 2020-10-12

**Authors:** Andriko Palmowski, Frank Buttgereit

**Affiliations:** Department of Rheumatology and Clinical Immunology, Charité – Universitätsmedizin, Berlin, Germany

**Keywords:** Glucocorticoids, Large vessel vasculitis, Adverse events, Takayasu arteritis, Giant cell arteritis, Prednisone, Steroids

## Abstract

**Purpose:**

While glucocorticoids (GCs) are effective in large vessel vasculitis (LVV), they may cause serious adverse events (AEs), especially if taken for longer durations and at higher doses. Unfortunately, patients suffering from LVV often need long-term treatment with GCs; therefore, toxicity needs to be expected and countered.

**Recent Findings:**

GCs remain the mainstay of therapy for both giant cell arteritis and Takayasu arteritis. In order to minimize their toxicity, the following strategies should be considered: GC tapering, administration of conventional synthetic (e.g., methotrexate) or biologic (e.g., tocilizumab) GC-sparing agents, as well as monitoring, prophylaxis, and treatment of GC-related AEs. Several drugs are currently under investigation to expand the armamentarium for the treatment of LVV.

**Summary:**

GC treatment in LVV is effective but associated with toxicity. Strategies to minimize this toxicity should be applied when treating patients suffering from LVV.

## Introduction

Large vessel vasculitis (LVV) is characterized by granulomatous inflammation of “large” vessels such as the aorta or its branches [1]. The two forms of LVV, giant cell arteritis (GCA) and Takayasu arteritis (TA), can lead to severe complications such as permanent loss of vision, strokes, or potentially lethal aortic aneurysms [2, 3].

Glucocorticoids (GCs) are the mainstay of LVV therapy, and instant administration attenuates LVV-associated morbidity by potent immunosuppression. Indeed, the anti-inflammatory and immunosuppressive properties of GCs are quasi-unrivalled.

However, GCs may cause complications themselves. Especially long-term treatment at higher dosages (> 5 mg/day; with a positive correlation between the daily and cumulative dose and the risk for adverse events) is potentially associated with adverse events (AEs) such as cardiovascular complications, osteoporosis, infections, cataracts, diabetes mellitus, weight gain, and cushingoid habitus. Unfortunately, patients suffering from LVV are usually in need of higher GC doses, particularly at the beginning of the disease and in case of higher disease activity. Also, the occurrence of relapses may cause the need for long-term treatment with GCs. Consequently, clinicians should expect GC-related toxicity in patients suffering from LVV and implement strategies that can reduce the frequency and severity of such AEs.

In this review, we will recapitulate the history of GCs in general, give insight into modes of action, and shed light on GC-related toxicity. We aim to provide strategies to clinicians in order to reduce this toxicity. Finally, we will have a look at ongoing research in this regard and future possibilities in the treatment of LVV.

## A Short History of Glucocorticoids

### The Discovery of Cortisone

In the 1930s, Edward Kendall succeeded in isolating a substance from the adrenal cortex, which he named “compound E”—later renamed as cortisone [4–6]. In September 1948, his colleague, Philip Hench, first administered cortisone to a patient suffering from rheumatoid arthritis [6]. This patient, a young woman of 29 years, who was previously bound to her wheelchair due to the crippling disease, experienced an unexpectedly powerful and rapid recovery [6].

### Glucocorticoids in Large Vessel Vasculitis

Horton was the first to administer GCs to a patient with GCA [7]. Further promising responses to this therapy were reported in 1957 by Birkhead et al. [8, 9]. Compared with the patients he had seen before, and compared with historical case reports from the literature, those treated with GCs were less frequently affected by progressive loss of vision, indicating that GCs could play a role in preventing GCA-associated complications [8, 9]. The first use of GCs in a patient with TA was described in 1954 [10]. However, treatment with GCs had to be discontinued due to an apparent worsening of the patient’s cataract—a now well-known GC-related AE. Three years later, in 1957, another author described a good response to GCs in a woman suffering from TA [11].

### The Discovery of Glucocorticoid-Associated Adverse Events

Initially, cortisone was considered a “modern miracle” (New York Times, 1949) [12]. Its discovery led to the only Nobel Prize in Physiology or Medicine that was ever awarded in the field of rheumatology [6]. However, as the lack of alternative, effective treatments for rheumatic diseases at that time led to extensive long-term use of GCs at high dosages. Physicians quickly started to recognise the variety of associated AEs only a few years after the discovery of cortisone [6, 13, 14]. Sprague et al., in 1950, published a first study on common undesired effects related to extensive GC use [14].

## How Do Glucocorticoids Work?

GCs affect all immune cells in the human body [15, 16]. They reduce the number of circulating T-lymphocytes, monocytes, macrophages, eosinophils, and basophils; lower the synthesis of pro-inflammatory cytokines such as interleukin (IL)-2 and interleukin-6, and increase the number of circulating neutrophils [15, 16]. Furthermore, GCs modulate vessel permeability and the expression of adhesion molecules in endothelial cells [15, 16].

### Genomic…

These manifold effects are mediated by two separate modes of action [17]. Even at the lowest doses, such as ≤ 5 mg/day prednisone equivalent, GCs exert slow genomic effects [18]. They form a complex with the cytosolic GC-receptor, and the hormone-receptor complex binds to specific DNA sites causing “transactivation”. This results in an increased synthesis of anti-inflammatory proteins such as IL-10, annexin-1, or inhibitors of nuclear factor Kappa B (NFκB) [15, 19]. Of note, transactivation is (possibly oversimplistically) responsible for most GC-associated AEs [17, 19, 20]. At the same time, the hormone-receptor complex inhibits pro-inflammatory transcription factors causing “transrepression”, e.g., inactivation of NFκB [17]. Genomic effects are subject to a “ceiling effect” at approximately ≥ 100 mg/day prednisone equivalent due to GC-receptor saturation [18, 20].

### …and non-genomic modes of action

At high dosages, GCs also exert rapid non-genomic effects [16, 17]. Non-genomic effects are mainly mediated by non-specific interactions with cellular membranes, specific interactions with membrane-bound GC-receptors, and further effects mediated by the cytosolic GC-receptor [16–18]. Compared with the genomic effects outlined above, non-genomic effects (a) appear more quickly (seconds-minutes) [15, 19], (b) come into play only at high dosages (beginning at approximately ≥ 30–100 mg/day prednisone equivalent) [17, 20], and (c) do not have such strong “ceiling effects” [18, 20]. Non-genomic effects are especially desired in acute situations, e.g., in anaphylaxis or complicated GCA [19].

## Glucocorticoid-Associated Adverse Events

In 1950, Sprague et al. already gave a comprehensive, although not complete, overview of common undesired effects related to extensive GC use including metabolic (weight gain), psychiatric (euphoria and depression), dermatologic (acne vulgaris, hirsutism, keratosis pilaris, and skin striae), and gynaecological (menstrual disorders) AEs [14].

### Glucocorticoid Dosages and Glucocorticoid-Associated Adverse Events

Generally, there is broad consensus in rheumatology that long-term therapy with ≤ 5 mg/day prednisone equivalent is associated with a low level of harm, and that long-term therapy with ≥ 10 mg/day prednisone equivalent may often cause clinically significant AEs [21]. Between 5 and 10 mg/day prednisone equivalent, the actual level of toxicity is thought to be determined by patient-specific factors (more information on this can be found below) [21].

### Evidence for Glucocorticoid-Associated Adverse Events in Large Vessel Vasculitis

Concerning LVV in particular, several observational studies have been published in recent years that investigated GC-associated AEs. A retrospective study in medical claims data found an overall rate of 0.43 AEs per patient-year of GC exposure in GCA [22]. The most common AE was cataract (0.16 events per patient-year), followed by bone disease (0.16 events per patient-year; including osteoporosis, fracture, hip replacement, and aseptic necrosis of the bone). However, this study could (by design) not analyse for common AEs such as weight gain. A study in US electronic health records found an increased risk of new-onset type 2 diabetes mellitus (absolute risk 13.7% over the first year among patients without a prior diagnosis of diabetes) and a worsening over time of pre-existing diabetes with a GC dose-response relationship, accompanied by an increase in body mass index [23]. A limitation of this study is the lack of a non-GCA control group.

On the other hand, a prospective cohort study that included outpatient GCA cases (all taking GCs at baseline) found a marked increase in the prevalence of osteoporosis (+ 12.6 percentage points over 3 years), but only a marginal increase in the prevalence of diabetes and hypertension over 3 years [24]. Of note, there were no healthy controls in this study and no dose-response analyses were carried out.

The risk of any type of infection in GCA is estimated to be around 0.16 per patient-year according to a UK study, and a dose-response relationship with GC intake has been suggested [25]. The most common infections in this study were lower respiratory tract infections, conjunctivitis, and herpes zoster [25].

### Causality or Correlation in Glucocorticoid-Associated Adverse Events

Randomized controlled trials usually find only a modest GC toxicity [26–28]. However, most trials are of short duration (usually ≤ 2 years) and are powered and designed to investigate efficacy rather than safety [29]. There is extensive observational research available concerning GC toxicity, but we want to point the reader to an important caveat here: Observational studies, by nature, cannot prove causality, and are subject to bias [30]. Not all AEs occurring during GC therapy are actually caused by GCs [31–34]. Observational studies, especially those investigating GCs, bear the risk of confounding by bias of indication: It is often the highly affected patients who are administered the highest GC doses, making it difficult to distinguish between treatment-related AEs and complications of the disease. For example, cardiovascular events may be the consequence of severe vasculitis or the consequence of intense GC therapy [33]. High disease activity usually also goes hand in hand with comedication that may contribute to AEs [29, 30, 34]. Additionally, age was shown to be positively associated with the risk of GC-related AEs [35], but higher age also increases the number of comorbidities and comedication. To put it in a nutshell, observational evidence on GCs must be interpreted cautiously.

### Attitudes of Patients and Physicians Towards Glucocorticoids and Glucocorticoid-Associated Adverse Events

Most patients suffering from rheumatic diseases acknowledge the positive effect that GCs have on their disease [36]. In two cohorts in Australia and the USA, only 2% and 8% of patients had the impression that GCs did not help “at all”, respectively, while 78% and 62% felt that GCs helped “a lot”, respectively [36]. The majority of these patients answered that the benefits outweighed the risks. However, the manifold potential AEs associated with long-term GC use represent a major topic for patients and physicians alike. In a study from 2009, osteoporosis was ranked the most worrisome AE by patients, followed by cardiovascular disease and diabetes mellitus [37]. For rheumatologists, diabetes stood on the top of the most worrisome AEs, followed by osteoporosis and hypertension [37]. In a qualitative study focussing on GCA and polymyalgia rheumatica patients, AEs associated with appearance (bruising, changes of facial contours, and weight gain) and diabetes were found to be relatively common and of major importance to patients [38]. Of note, the ambivalent position of medical professionals towards GCs—“fluctuating between strong endorsement and pronounced rejection” [29]—was seen as an additional burden for patients [38].

## Glucocorticoid Treatment in Large Vessel Vasculitis

Current guidelines concerning the clinical management of LVV are available from the European League Against Rheumatism (EULAR; for both GCA and TA) [39••], the British Society for Rheumatology (BSR; for GCA) [40], and the Swedish Society of Rheumatology (SSR; for GCA) [41].

### Initial Treatment

Initial treatment of uncomplicated LVV should consist of early high-dose oral GCs (40–60 mg/day prednisone equivalent), while an initial pulse therapy with intravenous GCs (0.25–1 g methylprednisolone) can be considered in patients with ischemic complications such as acute loss of vision or amaurosis fugax (“complicated” GCA). The risk of toxicity due short-term GC therapy is regarded to be relatively low.

### Glucocorticoid Tapering in Rheumatic Diseases

There is broad consensus that GCs should be tapered in rheumatic diseases if disease control has been achieved. In a population-based study, the cumulative incidence of GC-related AEs was similar in patients with and without polymyalgia rheumatica after 5 years [42], and the authors attribute this fact to the low GC doses, both daily and cumulative, achieved in the cohort, underpinning the importance of GC tapering. Indeed, the clinician must always weigh the risk of GC-related AEs against the risk of relapse. Generally, the GC dose should be (a) as high as necessary, but as low as possible, and (b) GCs should be administered as long as necessary, but as short as possible.

### Glucocorticoid Tapering in Large Vessel Vasculitis

Guidelines for management of LVV and GCA uniformly recommend that GCs should be tapered to minimize GC toxicity once disease control has been achieved. The EULAR recommendations for LVV, for example, suggest (a) within 2 to 3 months: a dose reduction to 20 mg/day prednisone equivalent by reducing the daily dose by 10 mg every other week; and (b) within a year: a reduction to ≤ 5 mg (GCA) or ≤ 10 mg (TA). The BSR and SSR guidelines only provide exemplary tapering schedules. There are two main reasons for this disparity: Firstly, high-quality evidence on tapering regimens in LVV is lacking. An observational study found that rapid tapering was associated with a higher risk of relapse [43], but the only randomized trial that compared two GC tapering strategies, also concluding that rapid tapering increases relapses in GCA, was actually designed to investigate the efficacy of tocilizumab. The comparison between different GC tapering protocols (26 weeks versus 52 weeks) was made by confronting the two placebo arms (TCZ) [44••]. A randomized trial is currently ongoing that investigates two different GC tapering regimens, namely long (over 52 weeks) versus short tapering (over 28 weeks; clinicaltrials.gov identifier NCT04012905). Secondly, detailed decisions concerning long-term GC therapy in LVV should be individualized according to various patient-specific factors.

### Patient-Specific Factors in Glucocorticoid Treatment

**Fig. 1:**
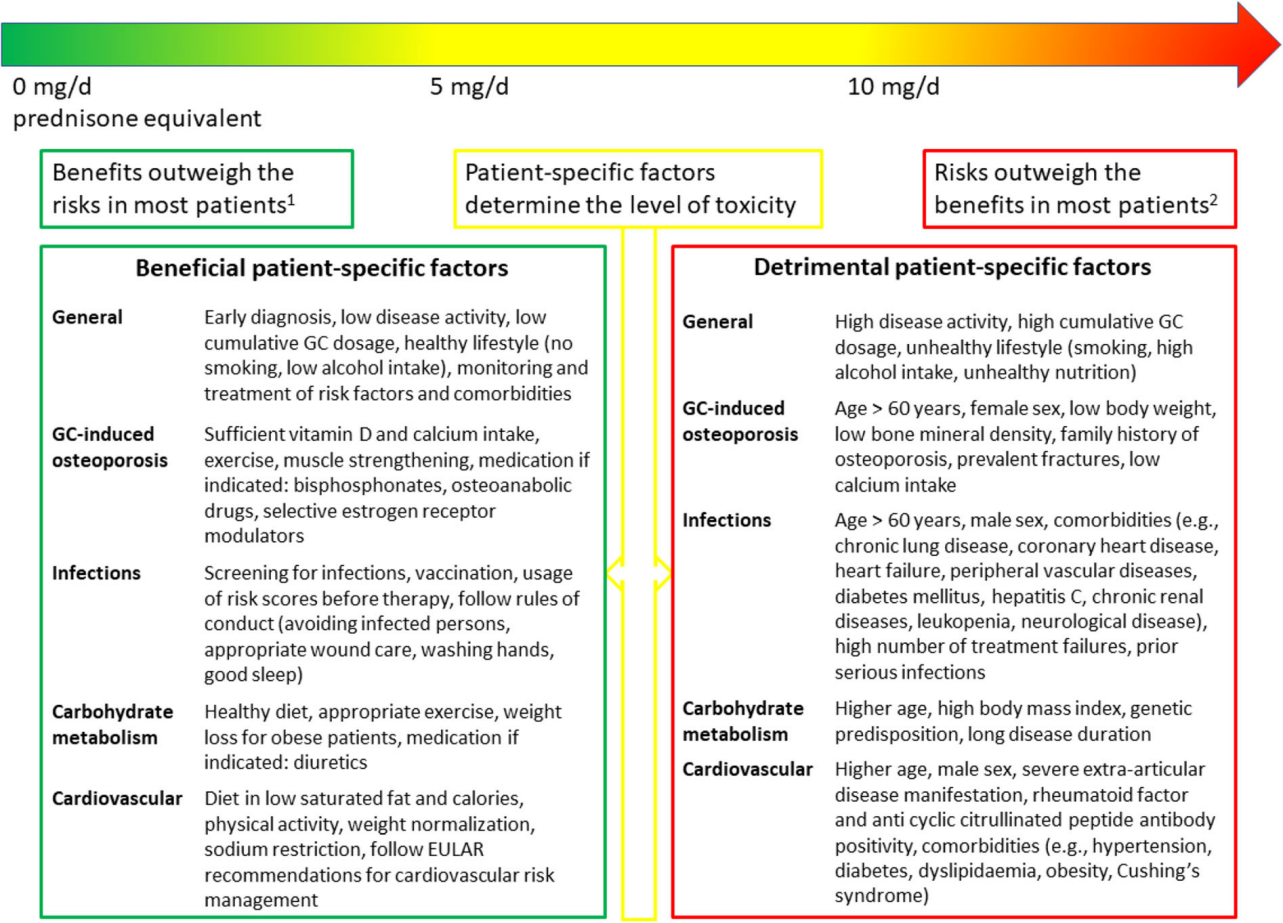
Patient-specific factors in long-term glucocorticoid therapy. GC, glucocorticoid. 1: not in patients with a high risk for cardiovascular events; 2: not in patients with glucocorticoid resistance. Adapted from: Cindy Strehl et al. *Defining conditions where long-term glucocorticoid treatment has an acceptably low level of harm to facilitate implementation of existing recommendations: viewpoints from an EULAR task force.* Ann Rheum Dis 2016;75:952–957

There are patient-specific factors that influence the benefit-risk ratio of GC treatment, e.g., age, comorbidities, or disease activity. In 2016, a EULAR task force conducted a systematic literature search and brought together clinicians, researchers, and patients to define conditions with an acceptably low level of harm in long-term (3 to 6 or more months) GC treatment [21]. In Fig. 1, we list factors that may increase or decrease the level of probable toxicity. These factors may guide the clinician concerning dose, duration, and tapering of GCs. Furthermore, patients’ preferences should be considered in the age of shared decision-making [20].

### Monitoring for Glucocorticoid-Associated Adverse Events

Patients that require long-term GC therapy should be monitored regularly for AEs, and EULAR has published recommendations for both low- and medium-/high-dose GC therapy [45, 46]. If disease control can be achieved by low-dose GCs, standard clinical care (including screening for hypertension) does not need to be extended except for osteoporosis and baseline assessments of ankle oedema and glucose intolerance. Screening for glaucoma should be performed if risk factors are present such as positive family history, high myopia, or diabetes [46]. In case of medium- to high-dose long-term treatment, monitoring for severe AEs that are treatable/preventable and common is recommended, provided that monitoring methods for these AEs are feasible and inexpensive [45].

### Prophylaxis of Glucocorticoid-Associated Adverse Events

Prophylaxis is possible for some GC-related AEs [47]. Osteoporosis should be countered by calcium and vitamin D supplementation if ≥ 7.5 mg/day prednisone equivalent is prescribed for more than 3 months [48]. Additional therapy with bisphosphonates can be considered depending on individual risk factors [48]. Non-pharmacological lifestyle modifications including weight-bearing exercise, cessation of smoking, and minimization of alcohol consumption should be recommended additionally [49]. For in-depth information on glucocorticoid-induced osteoporosis, the 2017 American College of Rheumatology guideline provides specific algorithms for both the prevention and treatment of this disease taking into account individual risk factors [49]. For example, it has been suggested to switch bisphosphonate treatment to another antifracture medication in adults who complete a planned oral bisphosphonate regimen but continue to receive GC treatment. The guideline also includes recommendations for specific populations (e.g., patients with organ transplants).

Generally, proton pump inhibitors should be administered in patients with concomitant use of non-steroidal anti-inflammatory drugs for gastroprotection. As the risk for infection is increased in patients with rheumatic diseases taking GCs, adequate immunization is advised, and patients should be instructed to seek help early in case of symptomatic infections [47].

### Treatment of Glucocorticoid-Associated Adverse Events

Patients suffering from hypertension, diabetes, dyslipidaemia, infections, and glaucoma can receive “standard” treatment with details given elsewhere [47]. Specific treatment of other GC-related AEs is reviewed elsewhere [47].

## Glucocorticoid-Sparing Agents in Large Vessel Vasculitis

In order to minimize GC toxicity, the GC dose should be as low as possible. Achieving a low GC dose in LVV should be facilitated by administration of GC-sparing agents that help to reduce disease activity and the risk of relapse in patients with significant comorbidity (e.g., diabetes, osteoporosis, or obesity), treatment-related AEs, or the need of long-term GC therapy. Attention should be given to the fact that treatment with GC-sparing agents may lead to AEs triggered by this additional therapy.

### Conventional Synthetic Glucocorticoid-Sparing Agents in Giant Cell Arteritis

Methotrexate (MTX) is the only conventional synthetic disease-modifying anti-rheumatic drug (DMARD) that has been proven to reduce the risk of relapse and the cumulative GC dose in GCA patients according to a high-quality systematic review and meta-analysis of randomized, placebo-controlled trials [50]. MTX, as an adjunctive therapy, is recommended in patients with refractory disease and in those who present with (or are at high risk for) GC-related AEs. A small trial also showed a modest benefit from azathioprine [51], and lower-quality evidence hints to a benefit from cyclophosphamide [52], leflunomide [53, 54], and dapsone [55]. Hydroxychloroquine and cyclosporin A were investigated in randomized trials but were not found to be effective GC-sparing agents in GCA [56–58]. Of note, all trials investigating GC-sparing agents in GCA have been unable so far to demonstrate a reduction of GC-related AEs.

### Biologic Glucocorticoid-Sparing Agents in Giant Cell Arteritis

The IL-6 receptor antagonist TCZ was investigated in two high-quality randomized controlled trials in GCA [44, 59]. It was shown that TCZ is able to reduce patients’ cumulative GC doses and increase the probability for sustained remission. Consequently, TCZ is recommended in selected patients with refractory disease and in those who present with (or are at high risk for) GC-related AEs (similar to MTX (see above)) [39••]. A randomized trial directly comparing TCZ to MTX will hopefully provide high-quality evidence as to whether one agent provides a better benefit-risk ratio (NCT03892785). Generally, one has to keep in mind that TCZ is a very expensive drug. Furthermore, monitoring of patients is challenging as TCZ suppresses the production of acute phase reactants such as c-reactive protein. Hence, monitoring disease activity in patients taking TCZ is largely symptom-based. Other biologic GC-sparing agents that have had promising effects are ustekinumab (an IL-12 and IL-23 inhibitor) [60], and abatacept (a T cell inhibitor) [61]; however, evidence concerning these two agents is sparse and of limited quality. Tumour necrosis factor α inhibitors (TNFi) were investigated in randomized trials, but they (infliximab, etanercept, and adalimumab) were found not to be effective for treating GCA [62–64].

### Glucocorticoid-Sparing Agents in Takayasu Arteritis

Generally, all patients suffering from TA should receive a GC-sparing agent due to high relapse rates and usually prolonged GC therapy [39••]. As TA is a rare disease, most evidence derives from observational studies with rather low quality. Initially, GCs should be accompanied by conventional synthetic DMARDs [39••]. Most authors suggest MTX as the first-line GC-sparing agent, although evidence is mainly based on low-quality studies and case reports. Alternatives are azathioprine, leflunomide, mycophenolate mofetil, and cyclophosphamide (reserved for patients with severe disease manifestations). In patients with relapse, TCZ (investigated in one of only four randomized trials in TA) or TNFi may be administered. TCZ showed clinical improvements and reduced relapse rates. While these results were statistically not significant, observational studies support the benefit of TCZ in patients with TA [65–68]. Interestingly, although randomized evidence on synthetic or biologic DMARDs is so scarce in TA, we found one randomized controlled trial each on curcumin [69•] and resveratrol [70•]. In these two short (≤ 3 months) trials, both curcumin and resveratrol lead to improvements of clinical (Birmingham Vasculitis Disease Activity Score) and laboratory (c-reactive protein and TNF) parameters. However, both studies did not report the use of GCs adequately and lacked appropriate description of AEs and thus results must be interpreted with caution.

## Outlook

GCs are still the treatment mainstay in GCA and TA. To reduce the toxicity that may arise from long-term GC use, TCZ has been approved for the treatment of GCA, and several agents are under investigation for both GCA and TA: New GC-sparing agents as well as new GC formulations might further reduce GC-related toxicity in LVV.

### New Glucocorticoid-Sparing Agents for Giant Cell Arteritis

Several agents are under investigation for treatment of GCA (Table 1). Sarilumab, another IL-6 receptor inhibitor, is currently under evaluation in a phase III trial. Furthermore, case reports on antibodies against IL-17 (secukinumab) and IL-1 (anakinra) have reported promising results [71, 72], leading to two randomized trials (phase 2 and 3, respectively) that are under way. Other potential agents under investigation are the Janus kinase (JAK) inhibitors upadacitinib and baricitinib, who have already been approved for rheumatoid arthritis. Pre-clinical research on JAK inhibitors had previously indicated that molecular signalling in LVV was dependant on JAK1 and JAK3 [73]. Granulocyte macrophage colony-stimulating factor was also shown to be upregulated in GCA tissue [74]; consequently, inhibition with mavrilimumab might provide relief in patients suffering from GCA.

**Table 1:** Ongoing randomized trials of glucocorticoid-sparing agents in giant cell arteritis and Takayasu arteritis

Disease	Experimental drug	Molecular target	Phase	Study start	Trial identifier	Target sample size (*n*)	Study population	Groups	Duration	Primary outcome
GCA	Sarilumab	IL-6-receptor α	3	11/2018	NCT03600805	360	New or refractory active disease	1: sarilumab dose A + prednisone 26-week tapering 2: sarilumab dose B + prednisone 26-week tapering 3: placebo + prednisone 26 weeks 4: placebo + prednisone 52 weeks	52 weeks of treatment + 24 weeks of follow-up	Sustained remission at week 52
	Secukinumab	IL-17A	2	1/2019	NCT03765788	50	New or refractory active disease	1: secukinumab + prednisolone 26-week tapering 2: placebo + prednisolone 26-week tapering	52 weeks of treatment + 8 weeks of follow-up	Sustained remission at week 28
	Anakinra	IL-1-receptor	3	5/2017	NCT02902731	70	New or refractory active disease	1: anakinra + prednisone tapering 2: placebo + prednisone tapering	52 weeks	Relapse rate at week 26
	Upadacitinib	Janus kinases	3	1/2019	NCT03725202	420	New or refractory active disease	1: upadacitinib dose A + corticosteroid 26-week tapering 2: upadacitinib dose B + corticosteroid 26-week tapering 3: placebo +52-week corticosteroid tapering	52 weeks	Sustained remission at week 52
	Mavrilimumab	Human granulocyte macrophage colony-stimulating factor receptor	2	12/2018	NCT03827018	60	New or refractory active disease	1: mavrilimumab + 26-week corticosteroid tapering 2: placebo + 26-week corticosteroid tapering	26 weeks of treatment + 12 weeks of follow-up	Time to flare at week 26
TA	Upadacitinib	Janus kinases	3	2/2020	NCT04161898	54	Refractory active disease	1: upadacitinib + prednisolone 2: placebo + prednisolone	Until 40 events of interest have occurred (approximately 31 months)	Time to first relapse
	Tofacitinib	Janus kinases	4	3/2020	NCT04299971	130	Active disease	1: tofacitinib + prednisone 2: methotrexate + prednisone	48 weeks	Remission at week 24
	Adalimumab	Tumour necrosis factor α	4	3/2020	NCT04300686	40	Severe active disease	1: adalimumab + prednisone 2: tocilizumab + prednisone	48 weeks	Remission at week 24

*GCA*, giant cell arteritis; *TA*, Takayasu arteritis; *IL*, interleukin

### New Glucocorticoid-Sparing Agents for Takayasu Arteritis

JAK inhibitors might be effective in TA, as well [73]. To date, evidence is limited to pre-clinical studies and a few case reports [75, 76], but two randomized trials are currently investigating the JAK inhibitors upadacitinib (phase 3) and tofacitinib (phase 4). Furthermore, a randomized phase 4 trial comparing adalimumab and tocilizumab has just been started to expand the evidence concerning TNFi treatment in TA.

### New Glucocorticoid Formulations for Large Vessel Vasculitis

The benefit-risk ratio of GCs in LVV might also be ameliorated by new GC formulations. Dissociated agonists of the GC-receptor (DAGRs) mainly trans-repress pro-inflammatory genes, while causing only little transactivation (which is thought to be responsible for the majority of GC-related AEs). In a trial of DAGRs in rheumatoid arthritis, fosdagrocorat 10 and 15 mg was as efficient as prednisone 10 mg with a safety profile similar to prednisone 5 mg [77••]. If further trials in rheumatoid arthritis support the use of DAGRs, investigations in LVV are surely warranted. Liposomal GCs are another option that might improve the benefit-risk ratio of GC therapy. The idea behind liposomal packaging is that GCs would mainly accumulate at the sites of inflammation, permitting high concentrations where needed while maintaining low systemic concentrations [6]. While this concept was promising, only little research has been conducted here within the last years: One of two trials (NCT00241982; “completed” in 2008) has only been presented at a rheumatology congress [78], while the other (NCT02534896) has reported some results on clinicaltrials.gov but is still awaiting publication in a peer-reviewed journal.

## Conclusions

GCs, although associated with toxicity in the long-term, remain the treatment of choice for patients with LVV. In order to minimize GC-related toxicity, several strategies are advised: GC dosages should be as low as possible but as high as necessary, and they should be prescribed only for the shortest possible time. Physicians should take patient-specific factors into account when evaluating the risk for GC-related AEs. GCs should be tapered, and GC-associated AEs should be countered by monitoring, prophylaxis, and adequate treatment. GC-sparing agents such as MTX or TCZ should be considered in patients with GCA and TA in order to achieve low GC doses. Several agents are currently under investigation to better treat LVV in future.
